# TMEFF2: A Transmembrane Proteoglycan with Multifaceted Actions in Cancer and Disease

**DOI:** 10.3390/cancers12123862

**Published:** 2020-12-21

**Authors:** Motasim Masood, Stefan Grimm, Mona El-Bahrawy, Ernesto Yagüe

**Affiliations:** 1 Department of Surgery and Cancer, Faculty of Medicine, Imperial College London, Du Cane Road, London W12 0NN, UK; m.masood14@imperial.ac.uk; 2 Department of Medicine, Faculty of Medicine, Imperial College London, Du Cane Road, London W12 0NN, UK; s.grimm@imperial.ac.uk; 3 Department of Metabolism, Digestion and Reproduction, Faculty of Medicine, Imperial College London, Du Cane Road, London W12 0NN, UK

**Keywords:** HPP1, TPEF, TENB2, tomoregulin, follistatin domain, circulating DNA, ectodomain shedding, interferon signalling, Alzheimer’s disease, epigenetic silencing

## Abstract

**Simple Summary:**

We recently came across an intriguing protein while screening for tumour-specific apoptosis inducers. It is known as the transmembrane protein with an EGF-like and two Follistatin-like domains 2 (TMEFF2). The gene was identified and characterized by five different groups almost simultaneously around 2000. Physiological function of TMEFF2 is elusive; however, the protein is reported to be involved in wide-ranging physiological and pathological functions including neuroprotection in Alzheimer’s diseases, interferon induction and one-carbon metabolism. Moreover, the *TMEFF2* promoter and 5′-upstream regions harbour a CpG island which is progressively methylated upon progression in a wide variety of cancers. Numerous primary publications suggest the methylation of *TMEFF2* as a prognostic and even diagnostic marker in different cancers. The primary literature regarding TMEFF2 is distributed far and wide, and despite having more than 150 primary publications mentioning TMEFF2 (or its aliases) in the title or abstract on PubMed, a comprehensive literature review is not available. We believe the reason behind this is firstly the sheer diversity of subjects of these publications and secondly the numerous primary publications reporting contradictory information about TMEFF2, especially when it comes to its oncogenic versus the onco-suppressive roles. The interest in TMEFF2 is growing again; PubMed returning at least 60 publications mentioning TMEFF2 (or its aliases) within the last year. We have made a laborious effort and written a comprehensive review article on TMEFF2 where we have not only compiled and contextualized the information regarding it but also critically analysed the information in the major primary publications. In addition, we have proposed some answers to the apparent TMEFF2 disagreements on its function. This information could serve as a valuable tool for readers not only about TMEFF2 but also on the dual role of type-I transmembrane proteoglycans (harbouring Follistatin-like domains) in oncogenesis and onco-suppression.

**Abstract:**

Transmembrane protein with an EGF-like and two Follistatin-like domains 2 (TMEFF2) is a 374-residue long type-I transmembrane proteoglycan which is proteolytically shed from the cell surface. The protein is involved in a range of functions including metabolism, neuroprotection, apoptosis, embryonic development, onco-suppression and endocrine function. *TMEFF2* is methylated in numerous cancers, and an inverse correlation with the stage, response to therapy and survival outcome has been observed. Moreover, *TMEFF2* methylation increases with breast, colon and gastric cancer progression. *TMEFF2* is methylated early during oncogenesis in breast and colorectal cancer, and the detection of methylated free-circulating *TMEFF2* DNA has been suggested as a potential diagnostic tool. The TMEFF2 downregulation signature equals and sometimes outperforms the Gleason and pathological scores in prostate cancer. *TMEFF2* is downregulated in glioma and cotricotropinomas, and it impairs the production of adrenocorticotropic hormone in glioma cells. Interestingly, through binding the amyloid β protein, its precursor and derivatives, TMEFF2 provides neuroprotection in Alzheimer’s disease. Despite undergoing extensive investigation over the last two decades, the primary literature regarding TMEFF2 is incoherent and offers conflicting information, in particular, the oncogenic vs. onco-suppressive role of TMEFF2 in prostate cancer. For the first time, we have compiled, contextualised and critically analysed the vast body of TMEFF2-related literature and answered the apparent discrepancies regarding its function, tissue expression, intracellular localization and oncogenic vs. onco-suppressive role.

## 1. Introduction

**Figure 1:**
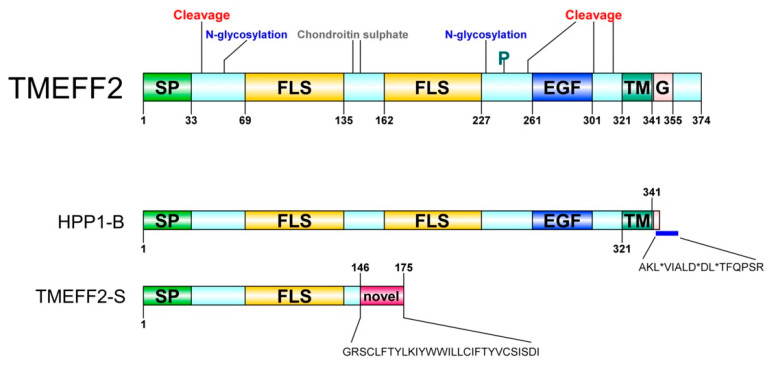
Transmembrane protein with an EGF-like and two Follistatin-like domains 2 (TMEFF2) protein architecture. SP: signal peptide, FLS: follistatin-like domain, EGF: EGF-like domain, TM: transmembrane domain, G: G-protein activating motif, P: phosphorylation site. A Ser/Gly rich region between the two follistatin-like domains constitutes a glycosaminoglycans (chondroitin sulphate) attachment site. A potential Tyr kinase phosphorylation site at residue 242, N-linked glycosylation sites around residues 55 and 203, and several potential protease cleavage sites (only shown cleavage sites reported through hydropathy analysis) are represented. HPP1-B is a less frequently isolated TMEFF2 variant originating from a 57-bp sequence insertion in its gene. The new sequence following the transmembrane domain contains three stop codons resulting in a truncated protein short of its cytoplasmic tail; stop sites are marked with *. TMEFF2-S is a short isoform where the first 146 residues of TMEFF2 are followed by a 29-residue long novel sequence. Imaging software: DOG [9].

The Transmembrane protein with an EGF-like and two Follistatin-like domains 2 (*TMEFF2*) gene is located on chromosome 2q32-q33 and encodes a 374-residue long single polypeptide, type-I transmembrane proteoglycan. According to the HUGO gene nomenclature committee [1,2], the aliases of TMEFF2 include HPP1, tomoregulin (TR), transmembrane protein TENB2 (TENB2), cancer/testis antigen family 120, member 2 (CT120.2), and transmembrane protein containing EGF and follistatin domains (TPEF). The reported functions of TMEFF2 span across a wide range of physiological and pathological spectra, ranging from corticotropin release hormone (CRH) stimulation in the anterior pituitary gland and providing neuroprotection by binding the amyloid β protein derivatives in Alzheimer’s disease on one hand to triggering the JAK-STAT pathway in colorectal cancer and modulating one-carbon metabolism in prostate tissue on the other. The *TMEFF2* promoter and its 5′-upstream CpG island [3] are methylated in a number of cancers. TMEFF2 is a multidomain protein with its N-terminus harbouring a signal peptide followed by two follistatin-like domains, an EGF (epidermal growth factor)-like domain, a transmembrane portion and a short intracellular domain (Figure 1). The EGF-like domain of TMEFF2 appears to be functionally ineffective because of the substitution of a crucial arginine residue (Arg39) with histidine [4], whereas the follistatin-like domains are reported to be crucial for the relevant functions of TMEFF2 [5,6,7,8]. Because of its implication in numerous cancers and involvement in diverse functions, TMEFF2 has attracted considerable interest since its identification in 2000; however, TMEFF2-related data appears inconsistent and sometimes conflicting across studies. Furthermore, TMEFF2 literature spans a variety of disciplines including neuroscience, cancer research, metabolism and extracellular matrix research. We have compiled and critically examined the salient information regarding TMEFF2 and offered answers to the apparent reported contradictions on its role and characteristics.

## 2. Identification and Characterization

TMEFF2 was identified and characterized by at least five independent groups within the time span of approximately a year [3,4,10,11,12]. A shorter secreted isoform of TMEFF2, TMEFF2-S, was reported later [13].

In April 2000, in an attempt to identify novel EGF-like genes, Akira Tanigami’s group [4] identified a novel gene expressed in the human brain after searching for the sequences encoding the conserved EGF-like domain and cloned it from a λ10 human foetal brain cDNA library. The predicted amino acid sequence revealed an N-terminal signal peptide, two follistatin-like domains upstream of the EGF-like domain, a putative transmembrane domain and a short C-terminal cytoplasmic domain. The gene was thus named *TMEFF2* (transmembrane protein with an EGF-like and two Follistatin-like domains 2). Later, the group isolated *Tmeff2* from a LambdaZAPII mouse brain cDNA library. The predicted amino acid sequences for human and mouse orthologs were 98.9% identical and showed a high degree of homology with the *X. laevis* protein x7365. A human clone, H7365 (identified in GenBank database, Accession number U19878), was found to share 35.8% homology with *TMEFF2* and was named *TMEFF1*. Gly and Cys residues typical of EGF-like domains are conserved in both TMEFF1 and TMEFF2; however, a critical residue, Arg39, which is crucial for the binding of the EGF-like domain to its receptors, is replaced with His in both TMEFF1 and TMEFF2. The mutation of this Arg in human EGF reduces its affinity for EGFR by 99.5% [4].

In a similar work, Satou’s and Sakamoto’s groups [11,14] cloned a cDNA from a human brain cDNA library while searching for novel proteins harbouring the EGF-like domain, which was named tomoregulin, and reported three splice variants: TRa (368-residues, TRb (418-residues) and TRc (379-residues). Hydropathy analysis of TRa revealed a G-protein-activating motif on the C-terminus, three protease cleavage sites—two adjacent to the transmembrane domain in the stalk sequence and another one towards the N-terminal portion of the EGF-like domain—two sites for N-linked glycosylation and two glycosaminoglycan attachment sites on the ectodomain. The experimental verification of N-linked glycosylation and the protease cleavage sites on both the N- and C-terminal portions of the EGF-like domain was also reported in this study [11,14].

Later, in October 2001, Robert Nicholson’s group [12] reported the discovery and characterization of *TENB2* while attempting to identify differentially expressed genes between androgen-dependent and androgen-independent stages of prostate cancer using the TEN12 prostate cancer xenograft model. The parent clone of the TEN12 xenograft is androgen-dependent and Nicholson’s group developed the androgen-independent TEN12-C and TEN-12-F by passaging the parent TEN12 through castrated and female mice. λcDNA libraries from TEN12 and TEN12-F were generated and screened, and *TENB2* was found among the cDNAs which were consistently upregulated upon development of androgen independence in the TEN12 prostate cancer xenograft. Interestingly, the authors described *TENB2* as a splice variant of tomoregulin [12] and observed a 100% sequence homology at the 5′-end of the two genes but not at the 3′-end region. Importantly, they reported that the isoform expressed in prostate tissue is exclusively *TENB2* and not tomoregulin. This was later contradicted by Harald Dinter’s group [15], who reported the expression of tomoregulin in prostate cancer and normal prostate tissue. In addition to the protein features described in the two previously mentioned studies, Nicholson’s group predicted a tyrosine kinase phosphorylation site preceding the EGF-like domain at residue 242 and an additional protease cleavage site at residues 40/41. Moreover, the group reported that the two follistatin-like domains in TENB2 exhibit 40–50% sequence similarity with the follistatin-like domains of other proteins. Nicholson’s group also demonstrated that the TENB2 protein was heavily glycosylated with both N-linked short oligosaccharides and O-linked long chondroitin sulphate (bimeric repeat polymer) glycosaminoglycans [12].

In September 2000, Peter Jones’s group [3] identified *TPEF* (transmembrane protein containing EGF and follistatin domains) during a search for the differentially methylated DNA fragments in colon, bladder and prostate cancers [16]. *TPEF* was mapped to human chromosome 2q33. Other salient features reported were as follows: (i) the promoter of *TPEF* is TATA-less and hosts a CpG island (−650 to +1 bp from the transcription starting point (tsp)), (ii) the most CpG rich region is −1100 to +750 from tsp, and (iii) the 3′-UTR includes a destabilizing ATTTA motif and two potential AATAAA polyadenylation signals followed by a polyadenylic acid stretch.

In December 2000, Barbara Leggett’s group [10] identified a novel gene, *HPP1*, that was differentially hypermethylated in colonic neoplasms and hyperplastic polyps. The cDNA was later cloned out using 3′-RACE (rapid amplification of cDNA ends) and showed 95% homology with *TMEFF2*, tomoregulin, *TENB2* and *TPEF*. Employing fluorescent in situ hybridization, *HPP1* was found to be located on chromosome 2 in region 2q32-q33. A second shorter isoform, *HPP1-B*, was also cloned and would encode a protein without the cytoplasmic tail. Interestingly, *HPP1-B* was only found in the normal mucosa and not in the cancerous tissues, but intriguingly, these investigators were unable to isolate tomoregulin from pooled normal mucosae by 3′-RACE.

Lastly, in 2006, Quayle and Sadar [13] reported a short isoform of *TMEFF2* (*TMEFF2-S*) in the LNCaP prostate cancer cell line. *TMEFF2-S* is comprised of the first four exons of *TMEFF2* followed by 2.9 kb corresponding to the fourth intron. The predicted amino acid sequence suggests a 175-amino acid open reading frame (ORF) consisting of the first 146 residues of TMEFF2 followed by a novel sequence of 29 mostly hydrophobic residues. TMEFF2-S lacks one follistatin-like domain, the EGF-like domain and the transmembrane domain. As the signal peptide was still intact, TMEFF2-S was found to be secreted in the medium of TMEFF2-S-GFP stable LNCaP cells. Notably, TMEFF2 was not observed in the media of LNCaP cells expressing the full-length TMEFF2-GFP fusion construct, suggesting that TMEFF2 is not a secretory protein in the proper sense (instead, it is released through proteolytic shedding—TMEFF2 in prostate cancer).

Regarding the proteolytic processing of TMEFF2, an important technical issue is the molecular masses reported on the immunoblots. The predicted molecular mass of TMEFF2 is 41,386 Daltons [12], although its reported values vary considerably, sometimes in the same cell lines and under same conditions (Table 1). We propose two factors as the cause of this variability. Firstly, TMEFF2 is a heavily glycosylated protein and, depending upon its glycosylation profile, the protein would travel in the SDS-PAGE at differing rates. Secondly, it is apparent from the studies by Vera Knauper’s group [5,17] that various proteases have the capacity to cleave TMEFF2 at different sites, resulting in several distinctive fragments for both the ectodomain and the remaining intramembranous stub. The prevalence of the specific protease moieties could in turn depend upon the cell/tissue type, microenvironment, cell culture conditions and cell transfection/treatment conditions. 

## 3. *TMEFF2* Promoter Methylation in Cancer

*TMEFF2* harbours a CpG island in its promoter region, spanning from −650 to +1 bp from the tsp [3]. Young et al. [10] RT-PCR analysis revealed that *HPP1* was expressed in 28/30 samples in normal mucosa but only 7/30 samples in cancer tissues. In the same study, loss of heterozygosity (LOH) analysis revealed that only 1/22 colorectal cancers, 3/6 adenomas and 1/22 hyperplastic polyps exhibited *HPP1* LOH. On the other hand, more than 30% DNA methylation was observed in 46/55 colorectal cancers, 6/9 adenomas and 17/27 hyperplastic polyps on 49 CpG sites spanning *HPP1* 5′-UTR and the first exon. A significant inverse correlation was also observed between the levels of *HPP1* promoter methylation and mRNA expression. Indeed, upon treatment of LoVo, HCT-116 [24] and HT29 adenocarcinoma cells with 5-aza-2-deoxycytidine (a DNA methyltransferase inhibitor), *HPP1* mRNA expression was restored, indicating that DNA methylation rather than LOH was the main contributing factor in silencing *HPP1* in colorectal cancer. Similarly, *TPEF* was found to be heavily methylated in 9 out of 11 cell lines tested by Liang et al. [3] and the mRNA expression was inversely correlated with *TPEF* methylation status. HCT15, LoVo, HCT-116, SW480, SW837, HT29, 3657 and DU145 cells harboured heavily methylated *TPEF* (>80%) and expressed almost no *TPEF* mRNA; SK-Mel-28 and LNCaP cells, on the other hand, showed less methylation (<20%) and expressed detectable *TPEF* mRNA levels [3]. A relatively recent study by Lee et al. [25] reported that high levels of *TMEFF2* mRNA were expressed by the cell lines with unmethylated alleles (H522, H1793, H2009 and H1703), while its expression was remarkably reduced in cell lines where the *TMEFF2* promoter was methylated (A549, H157, H226 and H1299). Again, the treatment of the non-expressing cells with 5-aza-2-deoxycytidine was able to significantly restore *TMEFF2* mRNA expression. An important confirmation of methylation being the primary source of *TMEFF2* gene silencing came from a gene-wide search by Young et al. for the somatic and germline mutations in colorectal cancer [26], where no pathogenic mutations in the *HPP1* gene from 63 tumours and the corresponding normal tissues were found.

A considerable number of primary publications have reported the differential *TMEFF2* methylation when compared to adjacent normal tissue or the corresponding tissue from healthy individuals in a variety of cancers including breast [27,28,29,30,31,32], prostate [3,31], lung [8,25,28,31], bladder [3], colon and rectal [3,8,10,24,28,32,33,34], gallbladder [35], renal [36], oesophageal [37,38], cardiac [37], stomach/gastric [10,37,39], ovarian [8], multiple myeloma [31], glioblastoma [8] and mesothelioma [40]. Several studies [3,8,10,24,25,39,41] among these are indicative of a direct correlation between *TMEFF2* mRNA downregulation and its promoter methylation, and some also reported promoter methylation to be related to the stages, response to therapy and survival outcomes of the corresponding cancers [8,27,28,31,39].

In breast cancer, *TMEFF2* promoter methylation increases progressively along the potential developmental path of invasive ductal carcinoma (IDC) from the preinvasive lesions like flat epithelial atypia (FEA), atypical ductal hyperplasia (ADH) and ductal carcinoma in situ (DCIS) [29,30]. Similarly, Young et al. [10] reported *HPP1* to be differentially hypermethylated in 84% of colon carcinomas, 66% of adenomas and 63% of premalignant hyperplastic polyps, again showing a progressive methylation profile during colorectal cancer progression [10]. In the case of ulcerative colitis-associated colorectal cancer, *TMEFF2* methylation was observed in 40% of dysplasia and 50% of carcinomas, but no methylation was detected in the non-neoplastic mucosae [24]. Sun et al. [42] presented similar findings in gastric cancer where TMEFF2 was notably expressed in 44.6% of normal gastric epithelium, 29.2% of intestinal metaplasia, 18.5% of dysplasia and 7.7% of gastric cancer. The group established an inverse correlation between TMEFF2 expression and its promoter methylation along the progression stages of gastric cancer and an association between low TMEFF2 expression and poor survival [41]. Together, these observations point towards a TMEFF2 onco-suppressive role.

Another interesting observation by Know et al. [29] is that *TMEFF2* methylation was higher in the normal tissue surrounding invasive breast cancer tissue from cancer patients as compared to the normal tissue from noncancer patients. Similarly, Sabbinioni et al. [43] reported that *TMEFF2* was methylated not only in colorectal cancer and the premalignant lesions but also in the tissue from the area around the premalignant lesions (adenomas). In a small cohort of 110 tumours and 21 normal breast tissue samples, the *TMEFF2* promoter was found to be hypermethylated in recurrent breast cancer compared to non-recurrent cancer (and normal breast tissue) [27]. In a subsequent study [28], Saraswati Sukumar’s group proposed *TMEFF2* methylation as a biomarker for the diagnosis of breast cancer in patients’ serum; *TMEFF2* was found to be among the top 10 out of 2674 genes differentially hypermethylated in the serum of breast cancer patients when compared to the sera from the healthy individuals. In colorectal cancer, Sabbinioni et al. [43] proposed the detection of methylated *TMEFF2* in blood as a diagnostic marker to identify patients with advanced colorectal cancer as well as with early tumorous lesions. Similarly, Herbst et al. [44] demonstrated the presence of methylated free-circulating DNA (mfcDNA) of *HPP1* as a prognostic marker, not only for the overall survival of the metastatic colorectal cancer (mCRC) patients but also as an early marker for the response of mCRC patients to combination therapy with fluoropyrimidine, oxaliplatin and bevacizumab. These studies clearly indicate *TMEFF2* methylation and subsequent silencing as early events during the development of breast and colorectal cancers.

The epigenetic silencing of *TMEFF2* is not limited to its promoter methylation. Want et al. [45] demonstrated restoration of *HPP1* expression in HCT-116 and DLD-1 colorectal cell lines upon inhibition and knockdown of histone deacetylase (HDAC). The *HPP1* promoter region harbours an E-box that is recognized by cMYC (a known repressor of *HPP1*). Want et al. [45] demonstrated that cMYC and HDAC3 colocalize at the *HPP1* promoter region and proposed a model where cMYC recruits HDAC3 to silence *HPP1* expression in colorectal cancer cells.

## 4. Tissue Distribution

TMEFF2 has probably not attracted much attention because of an apparently limited tissue distribution. Most studies report a baseline TMEFF2 expression exclusively in brain and prostate tissue [4,11,12,46,47]. However, numerous studies contradict this notion. For example, Liang et al. [3] used multi-tissue northern arrays and depicted *TPEF* to be predominantly present only in brain and prostate tissues, but a lower expression was observed in a variety of other tissues including stomach, colon, testis, ovary, pancreas, adrenal gland, thyroid gland, salivary gland, liver, small intestine, lung, trachea, placenta, foetal brain, foetal heart, foetal kidney, bladder, mammary gland, kidney and pituitary gland. Young et al. [10] employed in situ hybridization in colonic mucosae, and *HPP1* was observed in myofibroblasts, stromal cells, epithelial cells, B-lymphocytes, gut-associated lymphoid tissue, scattered fibroblasts, ganglion cells and endothelial cells. Using RT-PCR, Zhao et al. [15] observed low levels of tomoregulin mRNA in kidney tissue and minimal levels in testis. Han et al. [48] observed the expression of *TMEFF2* mRNA and protein in pancreatic cancer and normal pancreatic tissue. Intriguingly, Glynne-Jones et al. [12] used northern tissue arrays and reported *TENB2* mRNA present only in prostate tissue and brain (adult and foetal) except in the pituitary gland. On the other hand, Labeur et al. [6] found adrenocorticotropic hormone (ACTH)-producing cells of the anterior pituitary to be TMEFF2 immuno-positive.

The situation is similar when it comes to cell lines: most of the studies report no expression of TMEFF2 in the majority of cell lines except for a small number from prostate and nervous tissue origin [3,11,12,46,47]. Some important exceptions exist here as well. For example, Grey et al. [46] found low-level expression of TMEFF2 in HOS and MG63 osteosarcoma cells. Yonezawa et al. [11] reported weak expression in stomach fibroblasts, RD human embryonal rhabdomyosarcoma cells and PC12 rat pheochromocytoma cells in addition to the A172 glioblastoma and CHO-K1 cells. Han et al. [48] reported the expression of both the protein and mRNA of TMEFF2 in five pancreatic cancer cell lines—SW1990, Bxpc3, CFPAC1, Panc1 and AsPC1—and in the normal pancreatic ductal epithelial cells HPDE6-C7. Labeur et al. [6] used AtT20 pituitary corticotrope tumour cells for TMEFF2 knockdown studies. Moreover, Torrecilla et al. [23] cloned *TMEFF2* by retrotranscription from total RNA of the human lung carcinoma-derived cell line A549, and Liang et al. [3] used RNA from the human placental cDNA library for the same purpose. They also observed expression of the gene in the SK-Mel-28 human melanoma cell line pointing towards a more widespread tissue expression.

Interestingly, although several studies have proven the expression of TMEFF2 in prostate tissue [3,12,15,46,47], when Zhao et al. [15] tested for the expression of tomoregulin mRNA using RT-PCR and northern blot in five prostate cell lines (PrEC, BPH1, LNCaP, PC-3 and DU145), only LNCaP cells expressed significant levels of tomoregulin mRNA. The expression of TMEFF2 in LNCaP cells and its absence in PC3 and DU145 were also confirmed by Gery et al. [46], who additionally reported *TMEFF2* mRNA expression in the CWR22 prostate cancer xenograft. The most comprehensive study regarding the expression of TMEFF2 in normal and diseased tissue and cell lines was conducted by Afar et al. [47] using custom DNA microarrays followed by TaqMan™ analysis confirmation. They reported TMEFF2 expression to be exclusive in LNCaP and CWR22 prostate cancer cell lines in addition to the glioblastoma cell lines U118 and U187MG. However, a closer observation of the data provided by Afar et al. reveals a low yet detectable level of *TMEFF2* in breast, colon and lung cancer cell lines. This could be explained due to the relative expression levels of *TMEFF2* mRNA in brain and prostate tissues masking the data from other tissues.

Among other species, a similar TMEFF2 expression pattern has been reported, i.e., mainly in brain and nervous tissue [4,15,49,50] with a few interesting exceptions. Yonezawa et al. [11,14] observed tomoregulin immunoreactivity in gastric mucosa, mesenchymal cells and fibroblasts localized in the lamina propria of rat. Chen et al. [51] reported Tmeff2 expression in the white adipose tissue of mouse. They also registered significant *Tmeff2* expression in the mouse prostate using PALP staining in *Tmeff2* knockout mice where the first coding exon of *Tmeff2* was replaced with cDNA encoding the human placental alkaline phosphatase (hPLAP). However, when Afar et al. [47] used immunostaining with antibodies for the TMEFF2 ectodomain, they found the mouse prostate to be negative for Tmeff2. The absence of Tmeff2 in mouse prostate was also reported by Corbin et al. [49].

Harbouring a secretory signal peptide, the predicted transmembrane and the cytoplasmic domains, TMEFF2 is expected to be primarily localized on the plasma membrane. Indeed, Quayle et al. [13] observed full-length TMEFF2-GFP to be primarily localized at the plasma membrane of LNCap cells. However, other reports regarding the intracellular localization of TMEFF2 are not consistent. For example, Hyun-Seok et al. [7] reported TMEFF2 to exhibit a granular cytoplasmic profile rather than a membranous localization in MC65 neuroblastoma cells. In the 22Rv1 prostate cancer cell line, the TMEFF2 puncta can be observed mainly along the rim of the nuclei (through immunofluorescence) [21] and TMEFF2 has also been reported to colocalise with β-actin and α-tubulin in these cells [20]. Zhao et al. [52] observed TMEFF2 in the lysosomes of PC3 prostate cancer cells; as TMEFF2 lacks the typical cell-surface protein internalization motifs found in type I and II receptor proteins which canonically trigger their turnover through lysosomal degradation, the presence of a novel internalization signal in TMEFF2 has been suggested. A summary of the studies reporting intracellular TMEFF2 localization is presented in Table 2.

## 5. Proposed Functions

The physiological role of TMEFF2 remains largely elusive. *Tmeff2* knockout mice are born normal, maintain the Mendelian ratio and do not show any structural defects. However, they are smaller in size, fail to gain weight and die around weaning age (approximately 3 weeks) [49,51]. A selective summary of proposed TMEFF2 functions is provided in Table 3.

The conserved EGF-like domain and the two follistatin-like domains of TMEFF2 have attracted the attention of most studies. Although the substitution of Arg39 with His in the EGF-like domain strongly reduces its affinity for EGFRs, Yonezawa et al. [11] observed TMEFF2 mildly activating HER-4 receptors in MKN28 gastric cancer cells. This raises the question of whether a mutation in the EGF-like domain could change its functionality. This is unlikely; for example, Ali and Knauper [5] reported that the addition of a soluble TMEFF2 ectodomain to HEK293T cells led to an increase in ERK1/2 phosphorylation, an effect vanishing dramatically upon treatment with the HER1 inhibitor AG1478. On the other hand, the same study also reported that the treatment of HEK293T cells with a TMEFF2 mutant harbouring only the EGF-like domain failed to induce an increase in cell proliferation. Similarly, at least three additional studies [6,7,8] have reported that the deletion of the EGF-like domain of TMEFF2 had no effect on its reported function.

Turning the attention to the follistatin-like domain, follistatin was initially reported as an activin binding protein in the developing oocyte [57]. Activin is one of the 33 members of the transforming growth factor β (TGF-β) family known mainly for its role in augmenting the gonadotropin-releasing hormone (GnRH)-mediated release of follicle stimulating hormone (FSH) from the anterior pituitary. Follistatin was originally known to be a non TGF-β family member which could bind and inhibit the actions of activin. Later, it was discovered that follistatin is expressed by a number of tissues throughout the body and binds other members of the TGF-β family. In addition, it plays a crucial part during embryogenesis, and follistatin-null mice are smaller in size and die soon after birth due to impaired breathing [57]. However, the role of the follistatin-like domain in the follistatin module-containing proteins is not clear. One follistatin module-containing protein (osteonectin) has been reported to bind and neutralize both platelet derived growth factors (PDGFs) and vascular endothelial growth factor (VEGF) [8]. Similar to osteonectin, Lin et al. [8] reported that TMEFF2 could bind PDGF-AA both in solution and on the cell surface. They also showed this binding to prevent PDGF-AA from inducing cellular proliferation in NR6 fibroblasts. Furthermore, the ability of TMEFF2 to prevent PDGF-AA-induced cellular proliferation was abolished upon deletion of its follistatin-like domains. Huang et al. [54] likewise reported binding of TMEFF2 with PDGF-AA in HEK293T cells. In this interesting study, Tmeff2 was found to be specifically upregulated in cells of oligodendrocyte lineage during the differentiation stage. As PDGF-AA had already been shown to prevent this differentiation, it was hypothesized that TMEFF2 could induce oligodendrocyte differentiation by binding and neutralizing PDGF-AA. However, this hypothesis was proven wrong as *Tmeff2* knockout mice did not exhibit any defects in oligodendrocyte differentiation. Also, the addition of TMEFF2 to the media containing HEK293T cells could not prevent PDGF-AA-induced phosphorylation of ERK1/2. On the other hand, TMEFF2 overexpression in HEK293T cells itself caused ERK1/2 phosphorylation, pointing towards a pro-proliferative role. Similarly, the addition of recombinant TMEFF2 ectodomain to primary cultures of hippocampal and mesencephalic neurons was reported to enhance their proliferation in a dose-dependent manner [4].

Employing elaborate gene correlation and ontology analyses over multiple platforms (based on the correlation of gene expression in uterine corpus endometrial carcinoma), Gao et al. [58] suggested TMEFF2 to be involved in a diversity of functions; the notable ones include metabolism, embryonic development, cytoskeletal binding, extracellular matrix binding, chromatin binding and the interaction of RNA polymerase II with DNA. Similarly, wide-ranging signalling pathways were reported to involve TMEFF2, which include cGMP-PKG, Hippo, Hedgehog, MAPK and phospholipase D signalling. A closer look at the data indicates that the leading TMEFF2 function is related to neurons and nervous system physiology. The TMEFF2-related gene set comprises the cellular components of both axons and dendrites as well as the pre- and postsynaptic components, GABA signalling and ion channel complexes [58]. Conversely, TMEFF2 was reported to exhibit oncogenic properties in endometrial carcinoma (EC), where its expression increased gradually through clinical stages, nodal metastasis and de-differentiation of endometrial tissue [58]. In addition, knocking down TMEFF2 in endometrial carcinoma cells (Ishikawa) resulted in reduced cell proliferation, invasion and migration, inhibition of MAPK and PI3K-AKT pathways, and downregulation of the signature proteins of epithelial to mesenchymal transition (EMT). The mechanism behind TMEFF2-driven inhibition of MAPK and PI3K-AKT pathways remain unexplored. Similarly, Li et al. [59] implicated TMEFF2 in the development of drug resistance in ovarian cancer cells through its positive regulation by the long noncoding RNA TMPO-AS1 (TMPO-AS1 ⟞ miR-200c ⟞ TMEFF2).

On the other hand, when compared to adjacent normal tissue, *TMEFF2* mRNA was downregulated in pancreatic cancer (72 patients) [48], and an inverse correlation was observed between TEMFF2 protein in pancreatic cancer tissue and the clinical stage of the disease. The study also reported that TMEFF2 overexpression in AsPC1 and Panc1 pancreatic cancer cells (i) impairs cell proliferation both in vitro and in vivo (xenograft), (ii) decreases their invasion and migration capacities, and (iii) downregulates EMT signature proteins. These characteristics were attributed to suppression of the p38 MAPK signalling pathway by TMEFF2. The study also claimed that the experimental overexpression of TMEFF2 reduced phosphorylation of RAS, RAF, MEK and ERK in both AsPC1 and Panc1 cells. The onco-suppressive role of TMEFF2 in pancreatic cancer was also observed by Li et al. [60] (the study reports positive regulation of TMEFF2 by LINC01963, LINC01963 ⟞ miR-641 ⟞ TMEFF2). Fan et al. [61] presented similar findings, where TMEFF2 overexpression led to an increase in apoptosis and a decrease in proliferation, cell migration and invasion capacities of the lung cancer cell line A549.

Likewise, not only was TMEFF2 expression downregulated in glioma but also its mRNA levels decreased with increasing grade and worsening treatment outcomes [8]. In support of the onco-suppressive role of TMEFF2, Labeur et al. [6] reported that TMEFF2 protein levels decreased in cotricotropinomas. TMEFF2 overexpression also caused a marked decrease in proliferation of AtT20 human glioma cells when tested through both short-term (WST-1 assay) and long-term (soft agar colony formation) cell proliferation assays. Furthermore, TMEFF2 inhibited the production of adrenocorticotropic hormone (ACTH) in response to CRH (corticotropin releasing hormone) stimulation in AtT20 glioma cells; this action was demonstrated to be the result of TMEFF2-imparted action at multiple levels of CRHR1 signalling. Notably, while the EGF-like domain, the intracellular domain and transmembrane domains were dispensable, the follistatin modules were deemed essential for the ability of TMEFF2 to impart the abovementioned effects.

**Figure 2:**
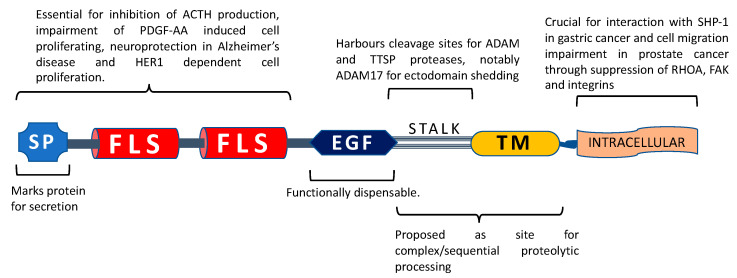
TMEFF2 domain functionality. SP: signal peptide, FLS: follistatin-like domain, EGF: EGF-like domain, TM: transmembrane domain. The N-terminal portion of TMEFF2 with signal peptide and follistatin-like domains is deemed crucial for impairment of adrenocorticotropic hormone (ACTH) production through inhibition of corticotropin release hormone (CRH) signalling in glioma cells, neuroprotection in Alzheimer’s disease, binding with PDGF-AA and induction of proliferation in HEK293 cells, whereas the EGF-like domain is dispensable for all of these functions. The stalk sequence (residues 303–320) is essential for ADAM17-mediated ectodomain shedding and harbours 2 potential cleavage sites for Type II transmembrane serine proteases (TTSPs). TTSPs like matriptase-1 and hepsin can cause TMEFF2 shedding independent of stalk region. Proteolytic processing of the ectodomain and remaining stub could impact TMEFF2 function. The TMEFF2 intracellular region is necessary for interaction with SHP-1 in gastric cancer cells and TMEFF2-induced impairment of cell migration in prostate cancer.

Another interesting role of TMEFF2 in the nervous tissue was reported by Hyun-Seok et al. [7]: TMEFF2 could bind amyloid-β protein oligomers (AβO) with high affinity and protected N2a cells from the neurotoxic effects of AβO. Amyloid-β protein (Aβ) self-aggregates into amyloid fibrils and causes amyloid plaque formation, which is a hallmark of Alzheimer’s disease. Amyloid-β protein is derived from proteolytic processing of the amyloid-β protein precursor (AβPP). In addition to fibrillar forms, amyloid-β protein exists in various smaller assemblies, called amyloid-β oligomers (AβO). TMEFF2 binding was not limited to the oligomers (AβO) but could directly bind the precursor AβPP and mature Aβ. Moreover, this binding was observed in solution, intracellularly and in mouse brain. Immunostaining revealed that Tmeff2 was upregulated in neurons exhibiting excessive amyloid-β intraneuronal accumulation, therefore suggesting a neuroprotective role of the protein in vivo. Considerable TMEFF2 immunostaining was also observed in astrocytes. In addition to binding and neutralization of AβO, the neuroprotective role of TMEFF2 was suggested to be the result of its neurotropic actions. MC65 neuroblastoma cell immunofluorescence showed TMEFF2 as a granular cytoplasmic appearance, suggesting its rapid internalization. ADAM17, the most notable protease responsible for TMEFF2 ectodomain shedding (discussed in detail in the following section—TMEFF2 in prostate cancer), also acts as the α-secretase for AβPP and cleaves it into a secreted form. Interestingly, overexpression of TMEFF2 in N2a cells enhanced the ADAM17-driven ectodomain shedding of AβPP. The cleavage site for ADAM17 in AβPP is located in the middle of the Aβ sequence, and consequently, AβPP ectodomain shedding by ADAM17 leads to a reduction in secreted Aβ. Hyun-Seok et al. [7] proposed a model where TMEFF2 could serve as a tether for ADAM17, leading to enhanced proteolytic processing of AβPP and thus reducing secreted Aβ. Once again, removing the EGF-like domain did not have any effect on the neuroprotective role of TMEFF2 (Figure 2).

## 6. TMEFF2 in Prostate Cancer

A substantial proportion of TMEFF2 literature focuses on its role in prostate cancer. Such studies greatly enhance the knowledge on TMEFF2, but at the same time, they impart perplexity about its function, the oncogenic vs. onco-suppressive nature of TMEFF2, the range of tissue expression as well as the possibility of TMEFF2 being a biomarker and/or a therapeutic target.

Numerous studies regarding the role of TMEFF2 in prostate cancer revolve around its progression from the androgen-dependent to androgen-independent stages. As mentioned earlier, Glynn-Jones et al. [12] identified *TENB2* while studying the differential gene expression between androgen-dependent and androgen-independent prostate cancer xenograft models. In addition, they also reported that TMEFF2 expression was markedly increased in prostate carcinoma vs. benign prostate hyperplasia and that its mRNA levels increased in higher-grade prostate cancers. These findings point towards an oncogenic role of TMEFF2 in prostate cancer. Such an inference is reinforced by several subsequent studies: for example, Bhaskar et al. [47] reported that both protein and *TMEFF2* mRNA levels increased significantly in prostate cancer compared to normal tissue. The authors also developed an antibody-drug conjugate (ADC) named Pr1-vcMMAE. Compared to the control ADC, Pr1-vcMMAE effectively reduced the tumour size in LNCap prostate cancer xenografts with minimal toxicity. The humanized version of ADC, hPr1-vcMMAE, gave similar results when used against the CWR22 prostate cancer xenografts. In what appears to be a follow-up study, Zhao et al. [15] used 2H8 tomoregulin mAb (developed by Uchida et al. [14]) radiolabelled with the β-emitting isotope ^90^Y and reported a significant size-reduction in the LNCaP xenografts (compared to radiolabelled IgG1). A substantial upregulation of tomoregulin mRNA and protein in prostate cancer tissue was also reported in this study. The injection of TMEFF2 mAb on its own did not have any effect on xenografts, and there was neither differences in proliferation reported in the tomoregulin-stable PC3 prostate cancer cells nor any difference observed in the proliferation of LNCap or PC3 cells when the extracellular domain of tomoregulin was added to the cell cultures. Similarly, Zhao et al. [52] complexed the TMEFF2 mAb (2H8) with a goat anti-mouse IgG antibody linked to the saporin toxin (Mab-ZAP). When added to TMEFF2-overexpressing PC3 prostate cancer cells (TMEFF2-PC3), Mab-ZAP induced a 50% reduction in the proliferation of TMEFF2-PC3 cells while the native PC3 cells remained unaffected. Again, TMEFF2-Stable PC3 cells showed no difference in cell proliferation compared to the control PC3 cells. These studies point towards TMEFF2 exhibiting oncogenic properties in prostate cancer, with higher expression in prostate cancer vs. normal prostate and further upregulation upon development of androgen independence. Moreover, ectopic overexpression of TMEFF2 had no detrimental effect whatsoever on the proliferation of prostate cancer cells in culture or in xenografts.

However, other studies point towards an altogether opposite function. Gery et al. [46] reported that TMEFF2 was expressed in androgen-dependent LNCap cells but not in the androgen-independent DU145 and PC3 prostate cancer cells. Moreover, reduced *TMEFF2* mRNA levels were observed in the androgen-independent prostate cancer xenograft models (LAPC9-AI, LAPC3-AI and LAPC4-AI) when compared to their androgen-dependent counterparts. Also, upon treatment of LNCaP cells with dihydrotestosterone (DHT), TMEFF2 expression increased in both time and dose-dependent manners. In addition, when an androgen-dependent prostate cancer xenograft model, CWR22, was propagated in castrated male mice and TMEFF2 expression vanished. Importantly, when TMEFF2 was ectopically expressed in DU145 and PC3 cells, it impaired their proliferation significantly; although TMEFF2 was upregulated by androgens, it exhibited antiproliferative effects in prostate cancer. Chen et al. [19] reported the steady state levels and the responsiveness of androgen receptors (AR) to have a direct correlation with TMEFF2 (mRNA and protein) expression in the xenograft derived from LNCap cells. Further corroboration of the onco-suppressive role of TMEFF2 in prostate cancer comes from the work of Georgescu et al. [62], who reported a positive correlation between high expression levels of *TMEFF2* mRNA and recurrence-free survival of prostate cancer patients. The study identified a set of 11 genes downregulated by TMEFF2 and designated them as TMCC11 (TMEFF2 modulated cell cycle 11). The native expression levels of TMCC11 increased upon treatment of LNCap cells with DHT, and endogenous TMEFF2 attenuated the DHT-driven upregulation of TMCC11, thus hampering the pro-growth effects of DHT in these cells. The study establishes high TMCC11 expression as a strong predictor of prostate cancer progression and recurrence, where C-statistics of the TMCC11 signature were reported to outperform the Gleason and pathological scores in one of the four datasets tested [62].

It is indeed intriguing to notice numerous prostate cancer studies reporting opposing data regarding the relation of TMEFF2 to androgens and its onco-suppressive versus potentially oncogenic role. A study by Mohler et al. [18] could offer an explanation for the former. In this study, the CWR22 androgen-dependent xenograft was implanted in the male host. After propagating the xenograft in male mice, the host was castrated, and the xenograft was left in the castrated host for 5 months (in order to find genes involved in the reoccurrence of prostate cancer). Eventually, an androgen-independent phenotype emerged which showed foci of highly proliferating cells. As expected, TMEFF2 was observed to be downregulated upon castration of the hosts. However, both *TMEFF2* mRNA and protein were upregulated in the androgen-independent CWR22 xenograft. Importantly, when two subsets of mice were treated with testosterone propionate at 6 and 20 days after castration, TMEFF2 was found upregulated upon androgen treatment. In conclusion, for these authors, *TMEFF2* is an androgen-regulated gene and its expression was abolished upon cessation of androgens, but it reappeared with even higher intensity in androgen-independent recurrent prostate cancer. This could, in part, offer an explanation for the apparent discrepancies in the TMEFF2 expression levels upon development of androgen independence in prostate cancer xenograft models. However, the marked expression of TMEFF2 in androgen-dependent LNCap cells and its absence in the androgen-independent DU145 and PC3 prostate cancer cells as well as the absence of TMEFF2 expression in the primary culture of human normal prostatic epithelial (PrEC) cells remain intriguing [15].

The conflicting oncogenic versus onco-suppressive role of TMEFF2 in prostate cancer literature is more complex to untangle. We will discuss some relevant studies and provide some possible explanations for these disagreements. The first study originates from María Ruiz-Echevarría’s group regarding the role of upstream open reading frames (uORFs) in the leader region of *TMEFF2* mRNA [21]. The uORFs are short open reading frames present upstream of the translation initiation sites in 5–10% of cellular mRNAs. uORFs can modulate the translation of mRNAs by hampering the translation of downstream coding regions, and more intriguingly, under cellular stress conditions, they can promote translation of the coding region. Under cellular stress, phosphorylation of eukaryotic translation initiation factor 2 (eIF2α) leads to a decrease in global translation, although the translation of certain genes (containing uORFs in their mRNA), such as *ATF4* (activating transcription factor 4), which is capable of initiating both pro-growth and pro-death signals [63], is enhanced [64]. *TMEFF2*, as *ATF4* mRNA, contains multiple uORFs. Echevarría’s group [21] established *Gaussia* luciferase (GLuc) constructs where *TMEFF2* 5′-uORFs were deleted one by one and placed under a cytomegalovirus (CMV) promoter. In both the androgen-dependent 22Rv1 prostate cancer cells and the androgen-independent PC3 cells, the removal of all four uORFs enhanced the translation of *TMEFF2* mRNA; however, when DHT was added to the 22Rv1 cell culture, a concentration-dependent increase in luciferase activity was only observed in the construct harbouring all four uORFs. Thus, although the uORFs in *TMEFF2* mRNA hampered the basal transcription of the gene, they were necessary for the androgen-dependent upregulation of TMEFF2. These authors also reported an enhanced eIF2α-dependent translation of TMEFF2 upon treatment with thapsigargin, a drug used to induce ER stress. This suggests that TMEFF2 is a stress-induced gene. There is a reasonable likelihood that, similar to other stress-related proteins, TMEFF2 could function as both an adaptive and apoptotic protein, which in turn would depend upon its expression level and/or posttranslational processing. The growth promoting vs. growth retarding effects of TMEFF2 in prostate cancer cell lines could therefore be decided by the degree of its expression and posttranslational modification state.

Vera Knauper’s group [5] actively searched for answers to the discrepancies between the oncogenic versus onco-suppressive roles of TMEFF2 in prostate cancer. The group specializes in investigating a family of membrane-bound and secreted metalloproteases known as ADAMs (a disintegrin and metalloprotease). One of the many functions of ADAMs is protein ectodomain shedding, a phenomenon where extracellular portions of membrane proteins are cleaved off by proteases and released into the surrounding ECM (or blood). In the case of the membrane bound ligands, chemokines and cytokines, ectodomain shedding leads to the release of the ligand from the cell surface, enabling them to interact with receptors in endocrine, autocrine or paracrine manners. A prime example of this phenomenon is the well-documented ectodomain shedding of the TNFα ligand by ADAM17 [65]. Owing to the previously reported ectodomain shedding of TMEFF2 upon treatment of A172 glioma cells with TNFα and interleukin-1 [66], Knauper’s group investigated TMEFF2 as a substrate of ADAM proteases. Upon treatment of TMEFF2-Stable HEK293T cells with phorbol ester α (PMA), the TMEFF2 ectodomain was released into the conditioned media. Moreover, the PMA-induced ectodomain shedding of TMEFF2 was either reduced or abolished upon inhibition of ADAM17. Importantly, TMEFF2 was shown to increase the proliferation of HEK293T cells both through overexpression and through the addition of soluble TMEFF2 ectodomain in the media. However, the simultaneous addition of ADAM17 inhibitor (TAP-1) and TMEFF2 overexpression reversed this increase in HEK293T cells proliferation, that is, the pro-proliferative effect of TMEFF2 was exclusive to its shed ectodomain. Ectodomain shedding can sometimes lead to activation of receptors through a phenomenon known as regulated intramembrane proteolysis (RIP). Here, after cleavage of the ectodomain, the remaining stub is cleaved within the membrane through an intramembrane-cleaving protease. The resulting fragment thus generated participates in subsequent signalling. Upon intramembrane cleavage of the Notch receptor, for example, the liberated intracellular domain is capable of entering the nucleus and of regulating the specific target genes [67]. Another important finding in this [5] and a subsequent study by Knauper’s group [17] was the phenomenon of processing the remaining membrane bound stub of TMEFF2 after its ectodomain was shed. TMEFF2 was demonstrated to be a substrate of several proteinases including γ-secretases, matriptase-1, matripase-2 and hepsin, with each enzyme being able to shed the TMEFF2 ectodomain (sometimes through combined sequential action) and, notably, cleaving the remaining membrane bound stub into specific fragments, distinct for each enzyme. Moreover, it was proposed that the fragments produced through the action of various proteases would function differently. The authors suggested it as a mechanism which would decide the oncogenic versus onco-suppressive activities of TMEFF2 depending on the enzymes available to process TMEFF2, which in turn would vary according to the cell type and/or the cellular and tissue microenvironment.

Ruiz-Echevarria’s group reported similar findings where overexpression of the full-length TMEFF2 impaired proliferation of HEK293T cells, sensitised the HEK293T cells for apoptosis by staurosporine and reduced colony formation in soft agar by approximately 5 fold [53]. In contrast, when only the TMEFF2 ectodomain was added to media of HEK293T cells and RWPE1 prostate cancer cells, it led to an increase in cell proliferation. Therefore, the intracellular domain would be necessary for onco-suppressive effects, whilst the shed extracellular domain would be a ligand enhancing cell growth. In addition, the overexpression of full-length TMEFF2-MYC-HIS in HEK293T cells was reported to bind and enhance the activity of sarcosine dehydrogenase (SRDH). Furthermore, a doxycycline-inducible TMEFF2-RWPE1 cell line was generated, and upon co-treatment with sarcosine and doxycycline, TMEFF2 reversed the cell migration enhancement effect of sarcosine. In a related study [20], Ruiz-Echevarria’s group observed that the TMEFF2-induced reduction in cell migration was not merely due to a decrease in the intracellular sarcosine concentration by enhanced SRDH but rather the metabolism of sarcosine was the key factor in this process, more specifically the one-carbon metabolism. One-carbon metabolism comprises a group of chemical reactions where one carbon group is transferred from one metabolite to another. During this process, a carbon carrier is needed, tetrahydrofolate being the most common one. Sarcosine and dimethylglycine are two of the main one-carbon donors in one-carbon metabolism pathways. When the antifolate drug methotrexate (MTX) was used, it abolished the increase in cell migration induced by TMEFF2 knockdown [20]. TMEFF2 knockdown also reduced the mRNA levels of dimethylglycine dehydrogenase (DMGH) and glycine decarboxylase (GLDC), which are important contributors to one-carbon metabolism.

Chen et al. [22] placed their emphasis on the role of potential GPCR signalling motif in the C-terminal portion of TMEFF2 as the putative deciding factor behind its onco-suppressive function in prostate cancer. The group cloned a deletion mutant lacking 13 consecutive basic-rich amino acids in the C-terminus of the protein (TMEFF2_ΔGA) and compared it to the full-length FL_TMEFF2. The overexpression of FL_TMEFF2 in RWE2 prostate cancer cells (i) reduced the expression of integrins αv, β1 and β3; (ii) inhibited RAS homologue family member A (RHOA) activation; (iii) impaired phosphorylation of the focal adhesion kinase (FAK); and (iv) promoted cell rounding, thus preventing lamellipodia protrusions leading to decreased migration of cells. On the other hand, the overexpression of TMEFF2_ΔGA failed to accomplish any of the above.

## 7. TMEFF2 in Gastric and Colorectal Cancers

Further corroborating evidence on the onco-suppressive role of TMEFF2 came from studies in gastric and colorectal cancers. As mentioned earlier, Sun et al. [41] reported significant downregulation of TMEFF2 through various stages of gastric cancer, which directly correlated with its promoter methylation and worse patient survival. This study identified five TMEFF2 interacting proteins in AGS gastric cancer cells, including tyrosine-protein phosphatase non-receptor type 6 (SHP1), Ras GTPase-activating protein-binding protein 1 (G3BP1), heterogeneous nuclear ribonucleoprotein K (HNRPK), splicing factor 1 and ubiquitin carboxyl-terminal hydrolase 4 (USP4). The interaction of TMEFF2 with SHP1 in AGS and MKN45 gastric cancer cells resulted in cell cycle arrest, induction of apoptosis and a decrease in cell proliferation both in vitro and in vivo (xenograft models). This interaction between TMEFF2 and SHP1 was abolished upon deletion of the TMEFF2 intracellular domain. Moreover, no mutations were found in the TMEFF2 intracellular domain in gastric cancer patients (TGCA data). These findings corroborate the previously mentioned importance of the TMEFF2 intracellular domain as well as the fact that promoter methylation rather than mutation is the primary cause of TMEFF2 loss of function. The investigators also proposed that TMEFF2 had a role in maintaining genomic integrity as TMEFF2 knockdown in GES-1 gastric cancer cells led to an increase in DNA damage. DNA damage-related genes were found to be enriched in TMEFF2 overexpressing AGS cells and gastric tumours with high TMEFF2 expression levels.

In a later study [42], the same research group investigated the role of TMEFF2 in *Helicobacter pylori*-induced gastric cancer development using cell cultures, mouse models and human gastric tissue pathology. *H. pylori* infection resulted in both upregulation and phosphorylation (activation) of STAT3, which repressed *TMEFF2* expression after binding to its promoter. On the other hand, TMEFF2 overexpression decreased STAT3 phosphorylation in AGS gastric cancer cells both in culture and in xenografts (TMEFF2 overexpression also resulted in a dramatic decrease in the tumour size). TMEFF2-induced downregulation of p-STAT3 was demonstrated to be dependent on its interaction with SHP-1.

Further evidence for the involvement of STATs in TMEFF2 function came from David Shibata’s group. Firstly, Elahi et al. [55] reported that TMEFF2 induced apoptosis in HCT-116 colorectal cancer cells and reduced their proliferation through activation of the STAT1 pathway. Interestingly, when TMEFF2-Stable HCT-116 colorectal cells were subjected to microarray analysis, 18 of the 42 upregulated genes were found to be interferon inducible. These interferon-inducible genes exhibited the highest fold change among the list of upregulated genes. The treatment of HCT-116 cells with STAT1 siRNA reversed TMEFF2-induced reduction of proliferation both on 2D and 3D cultures. In a subsequent study by the same group [56], it was reported that the TMEFF2 overexpression in HCT-116 cells led to upregulation of genes with both GAS and ISRE harbouring promoters (GAS to a higher degree than ISRE). Additionally, phosphorylation and subsequent nuclear localization of both STAT1 and STAT2 were observed in stably transfected TMEFF2 overexpressing HCT-116 cells. STAT phosphorylation is mediated by four kinases of the Janus family (JAK1, JAK2, JAK3 and TYK2), and TMEFF2 overexpression resulted in the upregulation of JAK1 and JAK2 but not the other two kinases. The HCT-116 cell line is resistant to interferon therapy (like several other cancers), and an interesting observation from this study [56] was that TMEFF2 overexpression marginally sensitized HCT-116 cells to INF-α but not INF-γ. In their latest study [68], David Shibata’s group observed the onco-suppressive and STAT1-inducing actions of TMEFF2 in HCT-116 cells to be dependent upon its proteolytic shedding (ADAM17 was identified as TMEFF2 shedase in HCT-116 cells).

## 8. Conclusions and Future Perspectives

Despite discrepancies, the overall literature points towards a context specific role of TMEFF2 when it comes to its oncogenic versus onco-suppressive function, with the latter predominating the former. Nevertheless, two factors should be taken into consideration while studying TMEFF2. Firstly, although functional, the expression levels of TMEFF2 in the majority of tissues and cell lines are very low. Even in prostate tissue where TMEFF2 is highly expressed, the inter- and intra-tissue variability is very high both in normal and cancerous tissue [15,62]. This aspect could affect the interpretation of data where the expression levels of TMEFF2 are assayed in tissue samples. Secondly, when it comes to studies involving cell lines, the cumulative evidence suggests that the extent and duration of experimental overexpression/knockdown could have significant effects on the outcome of these experiments. Similarly, for better consistency and reproducibility, cell culture and related transfection/transduction conditions should be factored in while interpreting the results of both past and future studies.

Two aspects of the posttranslational processing of TMEFF2 remain largely unexplored and are of interest in future studies. The first one is the phosphorylation of TMEFF2. Initial identification and characterization studies predicted one potential phosphorylation site on TMEFF2 just preceding the EGF-like domain [12]. However, the PhosphoSitePlus^®^ (PSP) database [69] reveals eight potential phosphorylation sites in TMEFF2 (Y202, T231, Y309, Y319, T358, Y361, S362 and S363). Differential phosphorylation could indeed have an impact on TMEFF2 function, especially under stress conditions. The second aspect of TMEFF2 posttranslational modification which remains to be investigated is its glycosylation. As the protein has both O-linked glycosaminoglycans and N-linked glycans attached, it could very well be possible that differential glycosylation of TMEFF2 affects its function.

In conclusion, *TMEFF2* is methylated in several cancers and could potentially be used as both a prognostic and a diagnostic marker. TMEFF2 is expressed in many tissues, the highest levels being observed in brain and prostate. The oncogenic vs. onco-suppressive function of TEMFF2 is decided by numerous factors including cell type, expression level, proteolytic processing and posttranslational modification state. Thus, TMEFF2 is an intriguing protein with multifaceted functions, the physiological role of which remains to be elucidated.

## Figures and Tables

**Table 1:** TMEFF2 immunoblot studies.

Cells	Reducing/Non-Reducing	Endogenous/Overexpressed	Antibody Used	Band Sizes kDa	Detail	Ref.
N2a	Not mentioned	Overexpressed	Anti-V5	17, 50, 56	Duplet band between 50 and 56 kDa, plus a single band just below 17 kDa (C-terminal band)	[7]
CWR22	Not mentioned	Endogenous	2H-8 ^a^	51, 58	Duplet band at 51–58 kDa	[18]
LNCap, C4-2	Not mentioned	Endogenous	TMEFF-CP ^b^	44	Single 44 kDa band in both LNCap and C4-2 cells	[19]
22Rv1, LNCap	Not mentioned	Endogenous	Abcam	~ 48	Single band just under 50 kDa marker (size not mentioned in text)	[20]
22Rv1, LNCap	Reducing	Endogenous	Abcam	40 to 55	Single smear ranging from 40 to 55 kDa in LNCap and doublet band between 40 and 55 kDa in 22Rv1 cells	[21]
RWPE1, RWPE2	Not mentioned	Overexpressed	Abcam	40 to 56	A smear from 40–56 kDa in RWPE1 and RWPE2 cells, a heavily glycosylated band can be observed above 90 kDa in only the TMEFF2_ΔGA form	[22]
HEK293	Reducing	Overexpressed	11D1C1 ^c^	58	Full length at 58 kDa, ECD only at 54 kDa (bands appear as duplets)	[23]
PC3, LNCap	Non-reducing	Both (see detail)	2H-8	43, 48, 52, 97	Endogenous TMEFF2 was detected at a single band of 43 kDa in LNCaP cells. V5/His tagged TMEFF2 overexpressing PC3 cells showed a major band at 48 kDa, a minor one at approximately 52kDa and another (smear) band at approximately 97 kDa. ECD-only overexpressing PC3 cells showed a band at 40 kDa.	[15]
CHO	Reducing	Overexpressed	Anti-V5	14, 71	V5/His tagged overexpressing TMEFF2 in CHO cells showed two distinct bands: one at 71 kDa and another at 14 kDa. For ECD-only overexpressing CHO cells, a duplet band at 54–60 was detected in cell lysate while 4 bands were detected in the medium, at 99, 69, 63 and 52 kDa.	[12]
CHO, A172	Both (see detail)	Both (see detail)	2H-8, aTR-C ^d^	51	In A172 cells, endogenous TRc was detected at 51 kDa under non-reducing conditions (2H8 antibody). For TRc-overexpressing CHO cells, the band was observed at 51 kDa under non-reducing conditions and at 58 kDa under reducing conditions in cell lysates (pAb-aTRc antibody). In the media of TRc-overexpressing CHO cells, under non-reducing conditions, a smear was observed at 39–45 kDa (2H8 antibody).	[11]
HEK293, LNCaP, CHO, PC3	Not mentioned	Both (see detail)	Anti-V5, Anti-TMEFF2 (details not mentioned)	60, 75, 10, 14, 22	Endogenous TMEFF2 from LNCap cells, the shed form from media, showed a duplet band at 60–75 kDa. In TMEFF2/V5/His overexpressing HEK293, a duplet band at 60–75 kDa was detected in conditioned media (atni-TMEFF2 antibody) and 10, 14 and 22 kDa distinct bands were detected in the cell lysate (anti-V5 antibody). Similarly, in TMEFF2/V5/His overexpressing CHO and PC3 cells, 10, 14 and 22 kDa c-terminal bands were detected in the cell lysate using V5 antibody.	[5]
HEK293	Not mentioned	Overexpressed	Anti-V5	15, 17, 20, 24, 28	Only C terminal bands were mentioned. In HEK293 cells stably transfected with AP-TMEFF2-V5/His, bands changed upon overexpression of various proteinases: (Matriptase-1) 24 and 28 kDa, and (Hepsin) 15 and 20 kDa. A 17-kDa band was observed without overexpression of any protease and was proposed to be a result of native ADAM activity.	[17]

^a^ 2H-8 mAb was generated against TR extracellular domain [11]. ^b^ TMEFF-CP pAb was generated against a.a 345–359 of TMEFF2 cytoplasmic domain [19]. ^c^ 11D1C1 mAb was generated against TMEFF2 extracellular domain by DNA immunization [23]. ^d^ aTR-C pAb was generated against a.a. 346–360 of TRc cytoplasmic domain [11].

**Table 2:** Studies reporting TMEFF2 intracellular localization.

Cells	Antibody or Detection Method Used	Intracellular Location	Ref.
PC3	Pr1#19	Membranous and punctate-cytoplasmic	[47]
PC3	2H8	Membranous and punctate-cytoplasmic upon internalization, co-localizes with acidic organelles	[52]
MC65	48G2	Perinuclear and punctate-cytoplasmic, not membranous	[7]
22Rv1	Abcam (details not mentioned)	Punctate-cytoplasmic along the rim of the nucleus when tested though immunofluorescence; membranous, cytoplasmic and nuclear in cell fractionation	[21]
HEK293T	Not mentioned	Membranous and diffused-cytoplasmic upon immunofluorescence but exhibits mitochondrial appearance when fusion constructs were used	[53]
22Rv1	Abcam (details not mentioned)	Membranous, cytoplasmic, nuclear; colocalization with β-actin and α-tubulin	[20]
LnCaP	GFP-fusion construct	Membranous and diffused-cytoplasmic for full length and punctate-cytoplasmic for short secreted isoform	[13]

**Table 3:** Studies reporting TMEFF2 function.

Function	Oncogenic vs. Onco-Suppressive	Disease or Process	Reference
TMEFF2 binds and inhibits PDGF-AA through its follistatin-like domains.	Onco-suppressive	Several cancers	[8]
TMEFF2 promotes survival of hippocampal and mesencephalic neurons in primary culture.	Neither but pro-growth	Neuronal development	[4]
Tomoregulin mildly activates erbB-4 receptors in MKN28 gastric cancer cells.	Oncogenic	Gastric cancer	[11]
TMEFF2 inhibits Akt phosphorylation and production of ACTH in response to CRH stimulation in AtT20 glioma cells. TMEFF2 decreases the proliferation of AtT20 cells.	Onco-suppressive	Glioma	[6]
(i) TMEFF2 is specifically upregulated in OPCs at onset of cell differentiation, but it is dispensable in the process. (ii) TMEFF2 weakly interacts with PDGFA but does not prevent the action of PDGFA on its receptor in HEK293T cells. (iii) TMEFF2 overexpression increases ERK1/2 phosphorylation in HEK293T cells.	Neither but pro-growth	Development	[54]
Tomoregulin mRNA is expressed in mouse embryo at E-11, increases gradually till E-15 and stabilizes through to E-17.	Neither	Development	[11]
TMEFF2 is regulated by androgens in prostate cancer and inhibits the proliferation of DU145 and PC3 prostate cancer cells.	Onco-suppressive	Prostate cancer	[46]
TMEFF2mAb conjugated cytotoxic drug Pr1-vcMMAE effectively reduced the tumour size in LNCap and CWR22 xenograft with minimal toxicity.	Potentially oncogenic	Prostate cancer	[47]
β-emitting isotope 90Y-labelled TMEFF2 mAB 2H8 effectively reduces LNCaP xenograft size and is well tolerated.	Potentially oncogenic	Prostate cancer	[15]
TMEFF2 is expressed on the cell surface, and it is internalized and recycled in lysosomes. TMEFF2 mAB is complexed with cytotoxin saporin, and it causes death of TMEFF2-Stable PC3 cells.	Potentially oncogenic	Prostate cancer	[52]
TMEFF2 binds amyloid-β protein, its precursor AβPP and the neurotoxic oligomeric forms of amyloid-β protein AβPOs in vitro and in vivo. TMEFF2 protects N2a cells from neurotoxicity induced by AβPO.	None	Alzheimer’s disease	[7]
TMEFF2 ectodomain shedding is triggered by TNFα, is brought about by ADAM10 and ADAM17, and increases proliferation of HEK293T cells. TMEFF2 causes phosphorylation of ERK1/2.	Neither but pro-growth	Prostate cancer	[5]
TMEFF2 is a substrate for membrane anchored serine proteases (TTSPs) matriptase-1 and hepsin.	Depends on proteolytic processing of TMEFF2	Prostate cancer	[17]
Full-length TMEFF2 reduces cell proliferation and sensitises HEK293T cells to apoptosis, binds and augments sarcosine dehydrogenase (SRDH) activity and reduces sarcosine levels, leading to inhibition of cell migration. The TMEFF2 ectodomain does the opposite.	Ectodomain: oncogenic Full-length: onco-suppressive	Prostate cancer	[53]
Full-length TMEFF2 (i) reduces the expression of integrins αv, β1 and β3; (ii) inhibits RHOA activation; (iii) inhibits phosphorylation of the focal adhesion kinase (FAK); and (iv) reduces cell migration. Deletion of putative G-protein domain prevents all of the above.	Onco-suppressive	Prostate cancer	[22]
TMEFF2 reduces invasiveness of 22Rv1 prostate cancer cells through modulation of one-carbon metabolism. TMEFF2 interacts with the cytoskeleton.	Onco-suppressive	Prostate cancer	[20]
HPP1 induces apoptosis and causes changes in morphology and reduction in cell proliferation in HCT-116 colorectal cells through activation of the STAT1 pathway.	Onco-suppressive	Colorectal cancer	[55]
HPP1 activates JAK-STAT interferon pathways to induce apoptosis and to reduce proliferation of HCT-116 cells. TMEFF2 overexpression marginally sensitized HCT-116 cells to INF-α-induced cell death.	Onco-suppressive	Colorectal cancer	[56]
TMFF2 decreases STAT3 phosphorylation through its interaction with SHP1 in gastric cancer. STAT3 directly binds to the TMEFF2 promoter and represses its transcription.	Onco-suppressive	Gastric cancer	[42]
TMEF2 binds SHP-1 in gastric cancer, induces cell cycle arrest and apoptosis and prevents DNA damage.	Onco-suppressive	Gastric cancer	[41]

## References

[1] Tweedie S., Braschi B., Gray K., Jones T.E.M., Seal R.L., Yates B., Bruford E.A. (2020). Genenames.org: The HGNC and VGNC resources in 2021. Nucleic Acids Res.

[2] HGNC Database, HUGO Gene Nomenclature Committee (HGNC), European Molecular Biology Laboratory, European Bioinformatics Institute (EMBL-EBI), Wellcome Genome Campus. https://www.genenames.org/.

[3] Liang G., Robertson K.D., Talmadge C., Sumegi J., Jones P.A. (2000). The Gene for a Novel Transmembrane Protein Containing Epidermal Growth Factor and Follistatin Domains Is Frequently Hypermethylated in Human Tumor Cells. Cancer Res..

[4] Horie M., Mitsumoto Y., Kyushiki H., Kanemoto N., Watanabe A., Taniguchi Y., Nishino N., Okamoto T., Kondo M., Mori T. (2000). Identification and Characterization of TMEFF2, a Novel Survival Factor for Hippocampal and Mesencephalic Neurons. Genomics.

[5] Ali N., Knaüper V. (2007). Phorbol Ester-induced Shedding of the Prostate Cancer Marker Transmembrane Protein with Epidermal Growth Factor and Two Follistatin Motifs 2 Is Mediated by the Disintegrin and Metalloproteinase-17. J. Biol. Chem..

[6] Labeur M., Wolfel B., Stalla J., Stalla G.K. (2015). TMEFF2 is an endogenous inhibitor of the CRH signal transduction pathway. J. Mol. Endocrinol..

[7] Hong H.S., Maezawa I., Petrlova J., Zhao X.Y., John C.V., Jin L.W. (2015). Tomoregulin (TMEFF2) Binds Alzheimer’s Disease Amyloid-beta (Abeta) Oligomer and AbetaPP and Protects Neurons from Abeta-Induced Toxicity. J. Alzheimers Dis..

[8] Lin K., Taylor J.R., Wu T.D., Gutierrez J., Elliott J.M., Vernes J.-M., Koeppen H., Phillips H.S., de Sauvage F.J., Meng Y.G. (2011). TMEFF2 is a PDGF-AA binding protein with methylation-associated gene silencing in multiple cancer types including glioma. PLoS ONE.

[9] Ren J., Wen L., Gao X., Jin C., Xue Y., Yao X. (2009). DOG 1.0: Illustrator of protein domain structures. Cell Res..

[10] Young J., Biden K.G., Simms L.A., Huggard P., Karamatic R., Eyre H.J., Sutherland G.R., Herath N., Barker M., Anderson G.J. (2001). HPP1: A transmembrane protein-encoding gene commonly methylated in colorectal polyps and cancers. Proc. Natl. Acad. Sci. USA.

[11] Yonezawa M., Uchida T., Wada K., Akamatsu T., Mizoguchi A., Sakamoto C., Tsukui T., Sinoki K., Satou J. (2000). A novel epidermal growth factor-like molecule containing two follistatin modules stimulates tyrosine phosphorylation of ERBB-4 in MKN28 gastric cancer cells. Gastroenterology.

[12] Glynne-Jones E., Harper M.E., Seery L.T., James R., Anglin I., Morgan H.E., Taylor K.M., Gee J.M., Nicholson R.I. (2001). TENB2, a proteoglycan identified in prostate cancer that is associated with disease progression and androgen independence. Int. J. Cancer.

[13] Quayle S.N., Sadar M.D. (2006). A truncated isoform of TMEFF2 encodes a secreted protein in prostate cancer cells. Genomics.

[14] Uchida T., Wada K., Akamatsu T., Yonezawa M., Noguchi H., Mizoguchi A., Kasuga M., Sakamoto C. (1999). A Novel Epidermal Growth Factor-like Molecule Containing Two Follistatin Modules Stimulates Tyrosine Phosphorylation of erbB-4 in MKN28 Gastric Cancer Cells. Biochem. Biophys. Res. Commun..

[15] Zhao X.-Y., Schneider D., Biroc S.L., Parry R., Alicke B., Toy P., Xuan J.-A., Sakamoto C., Wada K., Schulze M. (2005). Targeting Tomoregulin for Radioimmunotherapy of Prostate Cancer. Cancer Res..

[16] Liang G., Salem C.E., Yu M.C., Nguyen H.D., Gonzales F.A., Nguyen T.T., Nichols P.W., Jones P.A. (1998). DNA Methylation Differences Associated with Tumor Tissues Identified by Genome Scanning Analysis. Genomics.

[17] Gawel-Beben K., Ali N., Ellis V., Velasco G., Poghosyan Z., Ager A., Knauper V. (2018). TMEFF2 shedding is regulated by oxidative stress and mediated by ADAMs and transmembrane serine proteases implicated in prostate cancer. Cell Biol. Int..

[18] Mohler J.L., Morris T.L., Ford O.H., Alvey R.F., Sakamoto C., Gregory C.W. (2002). Identification of differentially expressed genes associated with androgen-independent growth of prostate cancer. Prostate.

[19] Chen Q., Watson J.T., Marengo S.R., Decker K.S., Coleman I., Nelson P.S., Sikes R.A. (2006). Gene expression in the LNCaP human prostate cancer progression model: Progression associated expression in vitro corresponds to expression changes associated with prostate cancer progression in vivo. Cancer Lett..

[20] Green T., Chen X., Ryan S., Asch A.S., Ruiz-Echevarría M.J. (2013). TMEFF2 and SARDH cooperate to modulate one-carbon metabolism and invasion of prostate cancer cells. Prostate.

[21] Overcash R.F., Chappell V.A., Green T., Geyer C.B., Asch A.S., Ruiz-Echevarria M.J. (2013). Androgen signaling promotes translation of TMEFF2 in prostate cancer cells via phosphorylation of the alpha subunit of the translation initiation factor 2. PLoS ONE.

[22] Chen X., Corbin J.M., Tipton G.J., Yang L.V., Asch A.S., Ruiz-Echevarría M.J. (2014). The TMEFF2 tumor suppressor modulates integrin expression, RhoA activation and migration of prostate cancer cells. Biochim. Biophys. Acta.

[23] Torrecilla D., Lozano M.V., Lallana E., Neissa J.I., Novoa-Carballal R., Vidal A., Fernandez-Megia E., Torres D., Riguera R., Alonso M.J. (2013). Anti-tumor efficacy of chitosan-g-poly(ethylene glycol) nanocapsules containing docetaxel: Anti-TMEFF-2 functionalized nanocapsules vs. non-functionalized nanocapsules. Eur. J. Pharm. Biopharm..

[24] Sato F., Shibata D., Harpaz N., Xu Y., Yin J., Mori Y., Wang S., Olaru A., Deacu E., Selaru F.M. (2002). Aberrant methylation of the HPP1 gene in ulcerative colitis-associated colorectal carcinoma. Cancer Res..

[25] Lee S.M., Park J.Y., Kim D.S. (2012). Methylation of TMEFF2 gene in tissue and serum DNA from patients with non-small cell lung cancer. Mol. Cells.

[26] Young J., Barker M., Fraser L., Walsh M.D., Spring K., Biden K.G., Hopper J.L., Leggett B.A., Jass J.R. (2002). Mutation searching in colorectal cancer studies: Experience with a denaturing high-pressure liquid chromatography system for exon-by-exon scanning of tumour suppressor genes. Pathology.

[27] Fackler M.J., Umbricht C.B., Williams D., Argani P., Cruz L.A., Merino V.F., Teo W.W., Zhang Z., Huang P., Visvananthan K. (2011). Genome-wide methylation analysis identifies genes specific to breast cancer hormone receptor status and risk of recurrence. Cancer Res..

[28] Fackler M.J., Lopez Bujanda Z., Umbricht C., Teo W.W., Cho S., Zhang Z., Visvanathan K., Jeter S., Argani P., Wang C. (2014). Novel methylated biomarkers and a robust assay to detect circulating tumor DNA in metastatic breast cancer. Cancer Res..

[29] Park S.Y., Kwon H.J., Lee H.E., Ryu H.S., Kim S.-W., Kim J.H., Kim I.A., Jung N., Cho N.-Y., Kang G.H. (2011). Promoter CpG island hypermethylation during breast cancer progression. Virchows Arch..

[30] de Groot J.S., Pan X., Meeldijk J., van der Wall E., van Diest P.J., Moelans C.B. (2014). Validation of DNA promoter hypermethylation biomarkers in breast cancer—A short report. Cell. Oncol..

[31] Suzuki M., Shigematsu H., Shames D.S., Sunaga N., Takahashi T., Shivapurkar N., Iizasa T., Frenkel E.P., Minna J.D., Fujisawa T. (2005). DNA methylation-associated inactivation of TGFβ-related genes DRM/Gremlin, RUNX3, and HPP1 in human cancers. Br. J. Cancer.

[32] Zhang C., Zhao H., Li J., Liu H., Wang F., Wei Y., Su J., Zhang D., Liu T., Zhang Y. (2015). The Identification of Specific Methylation Patterns across Different Cancers. PLoS ONE.

[33] Belshaw N.J., Elliott G.O., Williams E.A., Bradburn D.M., Mills S.J., Mathers J.C., Johnson I.T. (2004). Use of DNA from Human Stools to Detect Aberrant CpG Island Methylation of Genes Implicated in Colorectal Cancer. Cancer Epidemiol. Biomark. Prev..

[34] Wynter C.V., Walsh M.D., Higuchi T., Leggett B.A., Young J., Jass J.R. (2004). Methylation patterns define two types of hyperplastic polyp associated with colorectal cancer. Gut.

[35] Takahashi T., Shivapurkar N., Riquelme E., Shigematsu H., Reddy J., Suzuki M., Miyajima K., Zhou X., Bekele B.N., Gazdar A.F. (2004). Aberrant promoter hypermethylation of multiple genes in gallbladder carcinoma and chronic cholecystitis. Clin. Cancer Res..

[36] Chen E., Zheng F., Yuan X., Ye Y., Li X., Dai Y., Chen L. (2017). The effect of TMEFF2 methylation on the tumor stage and survival outcome of clear cell renal cell carcinoma. Cancer Biomark..

[37] Geddert H., Kiel S., Iskender E., Florl A.R., Krieg T., Vossen S., Gabbert H.E., Sarbia M. (2004). Correlation of hMLH1 and HPP1 hypermethylation in gastric, but not in esophageal and cardiac adenocarcinoma. Int. J. Cancer.

[38] Hadjinicolaou A.V., van Munster S.N., Achilleos A., Santiago Garcia J., Killcoyne S., Ragunath K., Bergman J.J.G.H.M., Fitzgerald R.C., di Pietro M. (2020). Aneuploidy in targeted endoscopic biopsies outperforms other tissue biomarkers in the prediction of histologic progression of Barrett’s oesophagus: A multi-centre prospective cohort study. EBioMedicine.

[39] Shibata D.M., Sato F., Mori Y., Perry K., Yin J., Wang S., Xu Y., Olaru A., Selaru F., Spring K. (2002). Hypermethylation of HPP1 is associated with hMLH1 hypermethylation in gastric adenocarcinomas. Cancer Res..

[40] Suzuki M., Toyooka S., Shivapurkar N., Shigematsu H., Miyajima K., Takahashi T., Stastny V., Zern A.L., Fujisawa T., Pass H.I. (2005). Aberrant methylation profile of human malignant mesotheliomas and its relationship to SV40 infection. Oncogene.

[41] Sun T., Du W., Xiong H., Yu Y., Weng Y., Ren L., Zhao H., Wang Y., Chen Y., Xu J. (2014). TMEFF2 deregulation contributes to gastric carcinogenesis and indicates poor survival outcome. Clin. Cancer Res..

[42] Sun T.-T., Tang J.-Y., Du W., Zhao H.-J., Zhao G., Yang S.-L., Chen H.-Y., Hong J., Fang J.-Y. (2015). Bidirectional regulation between TMEFF2 and STAT3 may contribute to Helicobacter pylori-associated gastric carcinogenesis. Int. J. Cancer.

[43] Sabbioni S., Miotto E., Veronese A., Sattin E., Gramantieri L., Bolondi L., Calin G.A., Gafa R., Lanza G., Carli G. (2003). Multigene methylation analysis of gastrointestinal tumors: TPEF emerges as a frequent tumor-specific aberrantly methylated marker that can be detected in peripheral blood. Mol. Diagn..

[44] Herbst A., Vdovin N., Gacesa S., Philipp A., Nagel D., Holdt L.M., op den Winkel M., Heinemann V., Stieber P., Graeven U. (2017). Methylated free-circulating HPP1 DNA is an early response marker in patients with metastatic colorectal cancer. Int. J. Cancer.

[45] Wang J., Elahi A., Ajidahun A., Clark W., Hernandez J., Achille A., Hao J.H., Seto E., Shibata D. (2014). The interplay between histone deacetylases and c-Myc in the transcriptional suppression of HPP1 in colon cancer. Cancer Biol. Ther..

[46] Gery S., Sawyers C.L., Agus D.B., Said J.W., Koeffler H.P. (2002). TMEFF2 is an androgen-regulated gene exhibiting antiproliferative effects in prostate cancer cells. Oncogene.

[47] Afar D.E., Bhaskar V., Ibsen E., Breinberg D., Henshall S.M., Kench J.G., Drobnjak M., Powers R., Wong M., Evangelista F. (2004). Preclinical validation of anti-TMEFF2-auristatin E-conjugated antibodies in the treatment of prostate cancer. Mol. Cancer Ther..

[48] Han H., Zhan Z., Xu J., Song Z. (2019). TMEFF2 inhibits pancreatic cancer cells proliferation, migration, and invasion by suppressing phosphorylation of the MAPK signaling pathway. Onco Targets Ther..

[49] Corbin J.M., Overcash R.F., Wren J.D., Coburn A., Tipton G.J., Ezzell J.A., McNaughton K.K., Fung K.-M., Kosanke S.D., Ruiz-Echevarria M.J. (2016). Analysis of TMEFF2 allografts and transgenic mouse models reveals roles in prostate regeneration and cancer. Prostate.

[50] Kanemoto N., Horie M., Omori K., Nishino N., Kondo M., Noguchi K., Tanigami A. (2001). Expression of TMEFF1 mRNA in the mouse central nervous system: Precise examination and comparative studies of TMEFF1 and TMEFF2. Brain Res. Mol. Brain Res..

[51] Chen T.R., Wang P., Carroll L.K., Zhang Y.J., Han B.X., Wang F. (2012). Generation and characterization of TMEFF2 mutant mice. Biochem. Biophys. Res. Commun..

[52] Zhao X.Y., Liu H.L., Liu B., Willuda J., Siemeister G., Mahmoudi M., Dinter H. (2008). Tomoregulin internalization confers selective cytotoxicity of immunotoxins on prostate cancer cells. Transl. Oncol..

[53] Chen X., Overcash R., Green T., Hoffman D., Asch A.S., Ruiz-Echevarria M.J. (2011). The tumor suppressor activity of the transmembrane protein with epidermal growth factor and two follistatin motifs 2 (TMEFF2) correlates with its ability to modulate sarcosine levels. J. Biol. Chem..

[54] Huang H., Teng P., Mei R., Yang A., Zhang Z., Zhao X., Qiu M. (2017). TMEFF2 is expressed in differentiating oligodendrocytes but dispensable for their differentiation in vivo. Sci. Rep..

[55] Elahi A., Zhang L., Yeatman T.J., Gery S., Sebti S., Shibata D. (2008). HPP1-mediated tumor suppression requires activation of STAT1 pathways. Int. J. Cancer.

[56] Hernandez J.M., Elahi A., Clark W., Humphries L.A., Wang J., Achille A., Seto E., Shibata D. (2015). The Tumor Suppressive Effects of HPP1 Are Mediated Through JAK-STAT-Interferon Signaling Pathways. DNA Cell Biol..

[57] Patel K. (1998). Follistatin. Int. J. Biochem. Cell Biol..

[58] Gao L., Nie X., Zheng M., Li X., Guo Q., Liu J., Liu Q., Hao Y., Lin B. (2020). TMEFF2 is a novel prognosis signature and target for endometrial carcinoma. Life Sci..

[59] Li H., Zhou Y., Cheng H., Tian J., Yang S. (2020). Roles of a TMPO-AS1/microRNA-200c/TMEFF2 ceRNA network in the malignant behaviors and 5-FU resistance of ovarian cancer cells. Exp. Mol. Pathol..

[60] Li K., Han H., Gu W., Cao C., Zheng P. (2020). Long non-coding RNA LINC01963 inhibits progression of pancreatic carcinoma by targeting miR-641/TMEFF2. Biomed. Pharmacother..

[61] Fan J.M., Zheng Z.R., Zeng Y.M., Chen X.Y. (2020). MiR-323-3p Targeting Transmembrane Protein with EGF-Like and 2 Follistatin Domain (TMEFF2) Inhibits Human Lung Cancer A549 Cell Apoptosis by Regulation of AKT and ERK Signaling Pathways. Med. Sci. Monit..

[62] Georgescu C., Corbin J.M., Thibivilliers S., Webb Z.D., Zhao Y.D., Koster J., Fung K.-M., Asch A.S., Wren J.D., Ruiz-Echevarría M.J. (2019). A TMEFF2-regulated cell cycle derived gene signature is prognostic of recurrence risk in prostate cancer. BMC Cancer.

[63] Pitale P.M., Gorbatyuk O., Gorbatyuk M. (2017). Neurodegeneration: Keeping ATF4 on a Tight Leash. Front. Cell. Neurosci..

[64] Harding H.P., Novoa I., Zhang Y., Zeng H., Wek R., Schapira M., Ron D. (2000). Regulated Translation Initiation Controls Stress-Induced Gene Expression in Mammalian Cells. Mol. Cell.

[65] Edwards D.R., Handsley M.M., Pennington C.J. (2008). The ADAM metalloproteinases. Mol. Asp. Med..

[66] Lin H., Wada K., Yonezawa M., Shinoki K., Akamatsu T., Tsukui T., Sakamoto C. (2003). Tomoregulin ectodomain shedding by proinflammatory cytokines. Life Sci..

[67] Blobel C.P. (2005). ADAMs: Key components in EGFR signalling and development. Nat. Rev. Mol. Cell Biol..

[68] Elahi A., Ajidahun A., Hendrick L., Getun I., Humphries L.A., Hernandez J., Shibata D. (2020). HPP1 Ectodomain Shedding is Mediated by ADAM17 and is Necessary for Tumor Suppression in Colon Cancer. J. Surg. Res..

[69] Hornbeck P.V., Zhang B., Murray B., Kornhauser J.M., Latham V., Skrzypek E. (2014). PhosphoSitePlus, 2014: Mutations, PTMs and recalibrations. Nucleic Acids Res..

